# End of the road? The career intentions of under-represented STEM students in higher education

**DOI:** 10.1186/s40594-022-00366-8

**Published:** 2022-07-30

**Authors:** Billy Wong, Yuan-Li Tiffany Chiu, Órla Meadhbh Murray, Jo Horsburgh

**Affiliations:** 1 University of Reading, Reading, RG1 5EX UK; 2 Centre for Higher Education Research and Scholarship, Imperial College London, Exhibition Road, London, SW7 2AZ UK

**Keywords:** Identity, Career aspiration, Under-represented groups, Gender, Ethnicity, Social class

## Abstract

**Background:**

The analogy of the leaky pipeline has been used to describe STEM education, with lower student diversity from compulsory to post-compulsory education and beyond. Although extensive research has explored the views and experiences of school-aged children about STEM, fewer studies have examined the career intentions of STEM students at university, especially those from under-represented backgrounds (e.g., racial/ethnic minority, women and working class students). This paper draws on a large qualitative study that interviewed 110 under-represented STEM undergraduates in the UK. We focus on students’ STEM career intentions and the likely directions of their post-degree trajectories, drawing on the lenses of science identity and Social Cognitive Career Theory.

**Results:**

Three pathways were identified. The first group plans to pursue a career in or from STEM. While social inequalities may persist, the potential impact of these challenges may be neutralised by the personal drive and passion of STEM career-oriented students, who seem committed to *drive into an STEM future*. The second group stated intentions for non-STEM-related careers, leaving the STEM pipeline. The reasons students gave for their imminent departure from STEM are the better financial reward on offer in some non-STEM sectors, especially in finance and business, as well as wider social inequalities and stereotypes. The third group was undecided, those who are uncertain or unclear about their futures. Students described a general lack of direction or clear career pathway, from a complete lack of career ideas to an overload of options.

**Conclusions:**

We conclude with a reminder that the STEM pipeline is far from secured or equitable, despite apparent progress in participation and representation. We reiterate the importance of fostering a diverse, inclusive and supportive learning environment that maximises the participation, strengths and potential of all students, especially those from under-represented backgrounds. While it is not uncommon for STEM students to pursue careers outside of STEM, we need to be wary that those who exit the STEM pipeline are not *forced off the road* by social inequalities and exclusions.

**Supplementary Information:**

The online version contains supplementary material available at 10.1186/s40594-022-00366-8.

## Introduction

In many countries around the world, there is growing recognition that the participation and attainment of students in Science, Technology, Engineering and Mathematics (STEM) is unequal, including in higher education (AdvanceHE, 2021). A plethora of research, especially in the last decade from the UK and US contexts, has highlighted experiential differences and inequalities for students from under-represented backgrounds, with a broader concern that STEM continues to be exclusive and elitist (e.g., Archer & DeWitt, 2017; Hanson, 2009; Wong, 2016). The dominant group in undergraduate STEM programmes in the UK is White British men (AdvanceHE, 2021), most likely from socioeconomically privileged backgrounds (Martin et al., 2020).

The analogy of the *leaky pipeline* has often been used to describe students’ participation in STEM, whereby different student groups—often the under-represented—gradually exit STEM education. While there are critiques of this metaphor (see Lykkegaard et al., 2019), we feel it offers an accessible way to visualise the loss of talents in STEM as students travel into higher education and beyond. It is important to highlight the different career pathways for those who are under-represented and the reasons for this, which, for us, is usefully represented by this pipeline metaphor. Although existing studies have explored the views and experiences of students toward STEM in the school context (e.g., Archer & DeWitt, 2017), less research has examined the career intentions of STEM degree students at university (Smith & White, 2019). This paper provides a qualitative insight into the career intentions of under-represented STEM students, highlighting their career aspirations, expectations and intentions after graduation. We focus on ethnic minorities, women and working class students at university, including how the intersections of gender, race/ethnicity and class impacted our under-represented STEM students. We identified three career intention pathways, namely, an STEM future, a non-STEM future, and an undecided future. We use Social Cognitive Career Theory (SCCT) and science identity theory to explore STEM students’ active negotiation of their future career plans, highlighting individual agency and structural barriers. We argue that despite apparent progress in STEM inclusion, the pipeline into STEM careers is far from secured, with inequalities and lack of secure career pathways still a stumbling block in the STEM road.

### Under-represented STEM students and their future pathways

In UK higher education there is an increasing emphasis on graduate employability and students developing professional skills to justify the value of degree programmes (Wong et al. 2022a). However, the transition from STEM degree to STEM career is not straightforward. There is an apparent paradox between the supply and demand of STEM graduates. Smith and White (2019) found that despite the high quantity of STEM undergraduates in the UK, there remain unfilled STEM vacancies. This challenges the discourse of a general skills shortage in STEM, but does highlight shortages in particular sectors, such as engineering. Furthermore, Smith and White (2019, p. 37) conclude that ‘STEM graduates are not more likely to enter graduate positions than those with degrees in other subjects’ and suggest that both the career choices of STEM graduates and the ways employers recruit are responsible for the composition of the STEM workforce. Thus, there is a need to understand the career intentions of STEM undergraduates, especially those from under-represented backgrounds, as their current participation in STEM higher education does not guarantee their involvement in the STEM workforce.

In March 2020, the UK government announced a major investment in STEM education, continuing long-running efforts to promote the uptake of STEM subjects and careers, particularly amongst women (Gov.uk, 2020). Yet, this aspiration has not yet materialised as under-represented groups continue to fall out of the STEM pipeline (Smith, 2011; Smith & White, 2019). An All-Party Parliamentary Group (APPG) inquiry was launched into the equity of the UK STEM workforce in November 2020, which highlighted that: ‘65% of the STEM workforce are White men’, ‘the STEM workforce has a lower share of female workers (27% vs. 52%) than the rest of the workforce’, and that with the exception of ‘workers with Indian ethnicity… People of other ethnic minorities tend to be under-represented in STEM’ (BSA, 2020, p. 6). Despite yearly progress which meant over a million women are now working in ‘core-STEM’ roles, women are still under-represented across the STEM sector (WISE, 2020).

It is increasingly popular for UK-based university students to undertake a work placement or internship, which can support their understanding and experience of the world of graduate work (Gbadamosi et al., 2019). In the US, Korte et al., (2019, p. 92) discuss in relation to engineering that learning to participate in ‘organizational work and social dynamics outside of what many considered to be “real” engineering work’ is a key part of STEM students transitioning into STEM professionals. However, not all students can access such career enriching opportunities. Students from under-represented backgrounds often lack the social networks or support to gain these opportunities, which can include unadvertised or unpaid experiences that would be unviable (Bathmaker et al., 2013). However, opportunities for non-STEM-related work experiences, especially in business and finance, are largely accessible in the UK due to the open-advertising of paid positions, which are also high-status and high-wealth careers that are popular amongst STEM graduates (Bosworth et al., 2013; Wakeham, 2016). Similarly, Binder et al. (2016) highlight that elite universities in the US often host structured recruitment fairs in finance, consultancy and technology, presenting these careers as achievable with high salaries, which can be particularly attractive for students who are unsure about their career pathways. In both the US and UK, careers in finance and business appear more widely accessible with paid graduate schemes, alongside clear routes for progression and job security (Shu, 2016; Wakeham, 2016).

The educational experiences of under-represented STEM students in higher education have been critically studied, especially in the US through the lenses of gender and ‘race’/ethnicity, including the intersection of multiple inequalities (e.g., McGee & Bentley, 2017; Ong et al., 2018). Increasingly, STEM education research recognises the importance of taking an intersectional approach to acknowledge how individual experiences and structural inequalities are shaped by multiple and intersecting identities (e.g., Ireland et al., 2018), especially in the context of disadvantage or discrimination. For instance, Blackburn (2017) surveyed 10 years of research on women in STEM (2013–2018) and found that women, particularly from racially minoritised backgrounds, often felt that they did not belong in STEM because of sexism, stereotype threat and concerns about fitting in, which negatively impacted their identity and their likelihood to pursue STEM careers. Likewise, Ong et al. (2011) reviewed 40 years of research on the factors that influence the retention, persistence, and achievement of minority ethnic women in STEM higher education. They also highlight a range of factors and barriers, including issues of identity, sense of belonging and racial microaggressions, as well as the role of family, peers, faculty, and societal stereotypes. Emerging literature from the UK highlights similar issues and concerns (e.g., Wong et al., 2022b). While these issues are not deterministic, they are actively negotiated and managed by students, for example, discussions of ‘stereotype management’ in McGee and Martin’s (2011) research on high-achieving Black mathematics and engineering students from Midwestern US universities. McGee (2016) recommends more structural approaches to addressing bias and discrimination in STEM, as relying on individual responses, such as grit and determination, are insufficient, even though sharing experiences can be helpful. To navigate this dynamic between structural barriers to under-represented students’ participation in STEM and their active navigation of whether or not to pursue STEM careers, we use Social Cognitive Career Theory and science identity theory to conceptually frame our analysis.

### Social Cognitive Career Theory (SCCT)

To understand students’ career intentions, we use the widely applied Social Cognitive Career Theory (SCCT) (Lent et al., 1994). Building upon Bandura’s (1986) social cognitive theory, SCCT is a conceptual framework that explores how people navigate academic choices and career development processes, focusing on: interests, self-efficacy, and outcome expectations, and how these are negotiated in context, whereby support and/or barriers are experienced. Rather than using ideas of fixed personal traits or variables to explore individual decision-making about careers, SCCT emphasises dynamic interactions between agentic people, their behaviour, and their contexts in which variables might have different weight at different times (Lent et al., 1994). For instance, a student may enter university with high interest in their STEM discipline and a future career in that field, but as they progress their outcome expectations and interest may change, and other careers opportunities that may arise. SCCT has been used to explore STEM career trajectories including middle/secondary schoolchildren’s interest in STEM (Nugent et al., 2015), project-based learning in STEM (Beier et al., 2019), differences on the basis of gender and ‘race’/ethnicity (Carpi et al., 2017), alongside in specific disciplines, such as engineering (Lent et al., 2005) and computing (Lent et al., 2008), as well as for working class or low-income students’ pursuit of postgraduate studies in general (Tate et al., 2014). The acknowledgement of both the impact of intersecting forms of structural inequalities and students’ active negotiation of different factors in context when making future career decisions, helps us to avoid overly deterministic approaches to acknowledging the barriers presented by gendered, raced and classed inequalities in STEM.

### Science identity

Under-represented students may lack confidence in themselves as a ‘STEM person’ due to a lack of representation and recognition from academics and in STEM more generally. In the US, Carlone and Johnson (2007)’s model of science identity recognises that a viable identity in science requires recognition by self and by others, especially in the interrelated dimensions of competence, performance and recognition. However, for women of colour, external recognition is often complicated due to inequalities of gender and race, as these students are often judged by the established scientific community (largely white men) against popular imaginings of ‘the scientist’ (also mostly white men). Carlone and Johnson found that students who are further away from the ‘norm’, namely, minoritised students, experienced a broader range of challenges or barriers in their STEM education.

Science identity theory is a popular theoretical frame within the literature, especially when analysing intersecting forms of inequality, for example, Avraamidou’s (2020) in-depth exploration of a young White Kurdish–Turk Muslim working-class heterosexual immigrant woman in Western Europe studying physics, and Rodriguez et al. (2019) exploration of Latina STEM students in the US. While much of this literature positions science identities as oppositional to students’ marginalised identities, Morton and Parsons (2018) explore Black women’s identities in STEM and highlight participants’ positive self-identifications as Black women meaning power, strength, and success. Similarly, Calabrese-Barton et al. (2013) acknowledge the importance of self-narration of one’s identity and the work that individuals do to negotiate their identities, or identity work.

Such science identities may be more or less fragile or robust, as acknowledged by McGee’s (2015) work on high-achieving Black students’ experiences in mathematics. McGee conceptualises such identities as having three components—motivation to succeed, coping strategies, dispositions associated with success—with the more fragile identities focusing on challenging negative stereotypes others may have of them, whereas more robust identities describe students with increased self-efficacy, who maintain their disciplinary passion and often see themselves as role models for others.

Science identity theory helps to conceptualise how students’ perceptions of themselves can be impacted by structural inequalities, making it more or less difficult to project themselves into a future STEM career. If one is not recognised as a ‘science person’ it can be more difficult to imagine oneself as a science professional and confidently plan one’s STEM career. In addition, students with more robust science identities may help them push through barriers or persist longer down the STEM road than peers with more fragile science identities.

To explore STEM students’ active negotiation of their career pathways, we use both SCCT and science identity theory to make sense of their career intentions, identities, future imaginings of careers, and ultimately career choices.

## Methods

This paper is based on a large qualitative study that explored university students’ identity development in STEM degrees. The aims were to provide a rich and relevant understanding of how under-represented STEM students negotiated their undergraduate degree journey, including their development of identity, sense of belonging and—the focus of this paper—their career intentions. Our broad research question is as follows: What career pathways do under-represented students in STEM degrees intend to pursue, and why?

Students were recruited from two medium-sized pre-1992 English universities, with international reputations in STEM research. Potential students were recruited through email invitations that detailed our project aims and selection criteria, namely, under-represented students in undergraduate STEM degrees. Our recruitment process included a broad definition of what under-representation means, including self-identification as women or non-binary, an ethnic minority and/or working class, LGBTQ +, experiencing any form of disability, including those who are neurodiverse and/or have specific learning difficulties (SpLDs). To begin, we sought permission from selected staff who teach undergraduate STEM modules to relay our recruitment message to their students. Interested students then signed up through a short survey, where students submitted their contact and background information, including details of their under-representation in STEM. Students were then contacted to book an online interview.

Data collection was conducted between summer and autumn of 2020 and involved semi-structured interviews with 110 STEM undergraduate students. Due to the coronavirus pandemic social distancing restrictions, all interviews were carried out online. Each interview began with a video introduction from the researchers to provide an overview of the project and the interview process. For better quality, all cameras were switched off during the recorded phase of the main interview. After the recording, the researchers restarted the video to formally conclude the interview and thank the participants for their involvement. The average time for each interview was 50 min. All interviews were audio recorded, transcribed verbatim and data anonymised for publication.

Students were asked to discuss their views and experiences of their STEM education, including their choice of study, sense of belonging and identity at university, perceptions of the typical student in their discipline, post-degree plans, as well as how being an under-represented STEM student may have shaped their experiences (see Additional file 1: Appendix). We wrote a reflective summary after each interview to record our general impressions, which formed our supplementary data. The researchers had no pre-existing relationships with the participants, and an e-voucher was provided to students as a token of appreciation.

Students from all levels of undergraduate study were recruited, from a spread of STEM disciplines including the biological sciences, computer science, engineering, mathematics, medicine and physics. A handful of applied sciences degrees, from building surveying to food science to zoology, were also recruited. Of the 110 STEM students, 78 are women 31 are men and one is non-binary. For ethnicity, 38 self-identified as White British and 72 as ethnic minorities. Although our participants identified with a range of ethnicities, we use broader terms to strengthen anonymity, such as Black British, British East Asian, British Mixed, British South Asian, International Asian and White British. Given our broad interpretation of under-representation in our recruitment, our participants also included 41 who identify as working class, 23 as LGBTQ + and 27 with a disability. Many students occupied more than one under-represented group, for instance, of the 20 ethnic minority men, seven were also working class, and all 11 White men were working class. From the 52 ethnic minority women, 15 were working class.

It is difficult to highlight all combinations of intersecting identities and forms of under-representation, particularly while maintaining anonymity. Thus, Table 1 provides an overview, and throughout our presentation of data we also include, where appropriate and relevant to the analysis, some aspects of interviewees’ identities (i.e., gender, ethnicity and class). This paper focuses on the career pathways that under-represented STEM students intend to follow.

**Table 1:** Student demographic background by gender, ethnicity, class and disabled status

Men (31)	Women (78)	Non-binary (1)
20 Ethnic minority	52 Ethnic minority	0 Ethnic minority
18 Working class	23 Working class	0 Working class
7 LGBTQ +	15 LGBTQ +	1 LGBTQ +
8 Disabled	18 Disabled	1 Disabled

Each interview transcript was analysed in *NVivo* software, following a coding framework that was produced after the authors independently coded three interview transcripts by relevant themes. The provisional codes were discussed and compared, and any differences on the application of codes were debated until a consensus was reached. The production of our coding framework involved an iterative process of data analysis, where we moved back and forth between the data and analyses through the comparison of data (Corbin & Strauss, 2014). A number of higher level themes were created that corresponded to the interview topics, including codes that captured how students discussed their post-degree plans (e.g., *anticipated challenges to succeed, career-related work experiences, career support, specific career plans, typical careers in own degree*).

Below, we discuss the career intentions of our under-represented STEM students in three pathways, from those who expect to *drive into an STEM future* to those who believe they have reached the *end of the STEM road*, as well as those who are at *a crossroads in STEM*. We include the raw student numbers and their percentages for contextual purpose, but our focus is on the qualitative data. A range of demographic backgrounds are represented in each pathway (e.g., by students’ gender, ethnic and class backgrounds).

## Results

### Drive into an STEM future

Around two-fifths (45/110, or 41%) of students in our study had clear intentions to continue with STEM careers. They studied a range of STEM degrees, including the applied sciences (9/12), the biological sciences (18/37), engineering (1/16), mathematics (2/10), medicine (8/9) and the physical sciences (7/26).

Just over a quarter (12/45) of students with an STEM career intention plan to work in academia, while the rest (33/45) indicated that their primary aspiration is to enter the STEM workforce in the discipline of their study. The 12 aspiring future academics study the applied sciences (*n* = 1), the biological sciences (*n* = 8) and the physical sciences (*n* = 3). These students said they had already thought about doctoral research as part of their plans to continue with a postgraduate degree. While half of them (6/12) also mentioned an alternative career plan, such as in commercial research and development, the other half were convinced that their future was in STEM academia. For instance, Odessa (White British middle class woman), a biology student, said:

I really want to become an academic researcher. Working in someone’s lab and then potentially, hopefully, one day, running my own lab. That’s my goal… I want to be a scientist. Even when I was quite young, that was what I wanted to do. Doing my course has made me realise that.

Some degrees provide accredited training and qualification into the STEM workforce, notably in medicine in which student career intentions are overwhelmingly to be a doctor, with one exception which we revisit later. Similarly, for those in the applied sciences with a specialised STEM degree, most students maintained their initial motivation to become a professional in their specific field. For example, life science student Danniel (White British middle class non-binary) spoke of their lifetime passion in nature and the environment:

I’d like to go into conservation biology because it’s… important to me… growing up, I spent a lot of time watching David Attenborough documentaries. It’s something I’ve always been interested in. Now more than ever, there’s a need for improved conservation practices.

Perhaps a surprise, only one of our engineering students, Deku (International Asian middle class man), stated a career intention to work in engineering, but even he expressed uncertainty about this pathway, because ‘a lot of my friends and a lot of my seniors… tend to associate [engineering]… with things like consultancy or finance [careers]’. This apparent deviation is revisited in the discussion. Similarly, only a few mathematics and physical sciences students wanted to work in STEM, with careers in data science or analysis the popular choices.

For STEM-career-oriented students, their intentions to pursue a career in STEM were rarely in question because of their interest in the discipline and commitment to achieve their dreams. Their science identities appear strong, even though for some this also required a level of resilience and self-confidence to counter negative remarks by others, particularly related to their under-represented identities (Carlone & Johnson, 2007). Such negative experience include (cis)sexism for women and non-binary students, and racism amongst minority ethnic groups (Blackburn, 2017; Wong et al., 2021). For example, as medical student Michael (British Black working class man) reflected:

I think the issue of race, whether it’s consciously or subconsciously, I think there will always be barriers there. But I don’t let those define me, in the sense that I think I know how to navigate those things. Maybe, I think there will be barriers there and I will encounter them as I come across, but I just try not to let them hinder my choices.

Similarly, Lakshani (British South Asian working class woman) appears already prepared that her background may put her at a disadvantage in her aspirations to be an academic. Yet, she was adamant that ‘it will completely not stop me from getting where I need to be… [even if it] might take me longer’. As Lakshani explains, ‘I’ve always associated research with biology. That was kind of the first thing that came into my mind before I even did the degree’. While social inequalities persist and were highlighted by some STEM-career-oriented students, this does not seem to drive them off the road. As Lent et al. (2005) argue in relation to SCCT, students actively negotiate barriers which do not necessarily dull their interest, self-efficacy and outcome expectations. Indeed, some anticipate or even accept that their STEM journey is bound to include additional barriers due to structural inequalities, such as racism, but can weather them if they have a strong science identity. This requires recognition by themselves and others, highlighting the importance of mentors and external modes of recognition (e.g., grades, work experience, and feedback from other students and teaching staff).

### End of the STEM road

Almost one in four (26/110, or 24%) students in our study stated no aspirations to pursue a career in or from STEM, thus exiting the STEM pipeline. These students study mathematics (6/10), engineering (6/16), the biological sciences (8/37) and the physical sciences (7/26). No students from medicine or the applied sciences stated their intentions to leave STEM. The most popular intended career pathway, mentioned by half of the students who planned to leave STEM (13/26, or 50%), was to work in finance, while the others expressed interest in consultancy work, the creative industries, the education sector, general office work, and entrepreneurship.

The main reason students gave for their imminent departure from STEM was the better financial reward that is on offer in some non-STEM sectors, especially in finance and business (see Wong, 2015). Students are aware that the skills and trainings of STEM graduates are often highly valued and sought after outside of STEM, with more lucrative salaries from multinational corporations. For some, an STEM degree becomes a steppingstone for a finance and business career. According to engineering student Meghan (Black British working class woman):

I have been considering finance because… [these] are big money careers… I’m trying to get a summer internship for next year in finance… I don’t really know much about the nature of the profession… it was mainly to do with the fact that it’s a good-paying job… it might as well be what’s most beneficial to me.

Similarly, engineering student Elif (White European middle class woman) explained that a high-paying job will enable her to be financially independent, ‘essentially, right now my goal is to be independent. And so, I need to earn enough money to be able to detach from my family and be able to provide for myself’. Elif explained that while she is still passionate about STEM, she must be realistic and pragmatic about her future, especially with regards to financial security, which appears easier to achieve in the world of finance when compared to the field of STEM. If the STEM pipeline is to be secured for all students, regardless of class and personal/family finances, clearer pathways are needed into secure well-paid employment, for example, through paid internship opportunities and graduate schemes, as is prevalent in finance and business. Such schemes help to manage students’ outcome expectations of different pathways, a key feature of SCCT approaches to analysing STEM career decisions. Perhaps such schemes could be considered in more STEM professions, whether facilitated by universities, government or STEM employers themselves to help provide clearer pathways to secure, well-paid employment in STEM.

Aside from higher economic returns, the conclusion of the STEM pathway also reflects a saturation or re-evaluation of their earlier interest. It is not unusual for career aspirations to evolve over time, even during undergraduate study, as students develop and enrich their experiences. Some students said they were inspired and intrigued by their work experiences outside of STEM, which prompted a gradual shift in interest about their future. A handful of students confessed their loss of love and passion in their STEM degree, but felt it was too late to change or disengage from their current study. Beatrice (Black British woman), for example, admitted her struggle to maintain motivation in her study of biology, because she realised her interest is now in psychology. She plans to undertake a psychology-related Masters, but this required, ‘getting through the [undergraduate] degree and then not looking back’. While students generally agreed that their departments are supportive of careers in academia, with lab experience and internships on offer, there were fewer opportunities and support from staff related to career opportunities outside of their discipline, especially those unrelated to STEM. Physics student Greg (White British working class man) said that the opportunities provided by his department were narrow, often focused on becoming a physicist. Greg’s career aspirations were broad, currently in the business sector, perhaps as an entrepreneur. He felt there was a lack of career advice for people like him, because tutors were more invested and adept in supporting aspiring academics. As such, Greg felt departmental support for students with non-physics-related career intentions was limited, a view which we revisit in the discussion.

As mentioned earlier and in existing literature (e.g., Ong, 2005), STEM students from minoritised backgrounds can experience additional challenges in their pursuit of an intelligible identity, which can contribute to their departure from STEM. Francesca (Black British working class woman), for instance, said that her lonely experience on her degree programme has convinced her to seek a career away from STEM to something that is more inclusive:

I had friends but I was alone… I spent a lot of lunch times sitting by myself … A lot of people found friends quickly, and I didn’t know why I didn’t find friends that quickly … maybe it was the added pressure of being the only black girl ... I felt like people didn’t know how to approach me.

Francesca also shared her experiences of racism, where peers have ‘made a joke about the stereotypical angry black woman and then apologised to me in my face’, which made her extremely conscious in that ‘I know I stick out like a sore thumb’. She is mindful of perceptions that ‘I might be the token girl that they admitted into the department, because they need their figures to be up, so perhaps I’m not smart enough’, although she seemed confident in her own ability. However, she is aware that ‘there are so few minorities in [her discipline] … [either] black female lecturers or black lecturers in general’, which made her realise that a career in her discipline would be more difficult as a Black woman. Like many others whose STEM journey is at end of the road, Francesca intends to work in finance and consultancy, for reasons of financial security as well as the hope of better inclusivity and diversity. Thus, for those exiting STEM, material concerns about pay, job security, and working conditions, particularly around structural inequalities were important, alongside lack of staff support within their departments when exploring careers beyond their discipline or academia.

### A crossroads in STEM

Over a third (39/110, or 35%) of our STEM undergraduates were unsure about their future pathways. Many are still exploring or have multiple ideas about their future, including careers in, from, and outside of STEM. Students from all disciplines are represented, including engineering (9/16), physical sciences (12/26), the biological sciences (11/37), mathematics (3/10), the applied sciences (3/12) and medicine (1/9). It is not unusual for students to enrol in higher education with limited ideas about their future. Undergraduate study often provides students with the time to experience and develop their interests. However, students who expressed career uncertainties existed across all undergraduate levels, from first to final year.

Students who are at an STEM career crossroads described a general lack of direction or clear career pathway, from a complete lack of career ideas to an overload of options. For instance, biology student Heather (British East Asian working class woman) said she has ‘looked at a couple of career options… [but] not sure what exactly I want to get into’. She explored ‘going into sort of technology and that industry in terms of software engineering’, but also ‘professional finance sector… like auditing, insurance and things like that’. She was also keen on ‘different areas of consulting’, as well as ‘NHS [National Health Service] scientist training programme’ and ‘being a patent attorney’. In short, she and others like her have interest in a wide range of careers but are undecided about any particular pathway.

On the other end of the spectrum, John (White British working class man) said his reason for studying mathematics was purely ‘out of interest’, despite his lack of awareness about ‘where that leads as a career’. He recognised that:

Coming from the background I come from, I’ve not really been exposed to a lot of people who work in degree environments… but I don’t really know what people with degrees do all the time.

John’s educational pathway in mathematics was interest-driven and supported by his family and school, who seem to adhere to the discourse of mathematics as a ‘good’ study option, even though John himself remains uncertain about his career pathway. At the time of the interview he had just been accepted onto a Masters in statistics, ‘with a view to hopefully being inspired towards a specific career’. He hoped ‘the extra year at university’ would allow him to ‘ask all the questions that I want to ask, to find out what I could do beyond sort of general careers skills, sort of now take an opportunity to speak to people’. For working class students, such as John, their engagement with STEM, including plans for postgraduate study, may be borne out of uncertainty and a lack of understanding about their future options. For John, further study means he can buy time and institutional support to build and strengthen his knowledge about possible career pathways.

Even amongst students with greater clarity about their career intentions, especially in STEM, we found expressions of uncertainties relating to self-identity. For example, physics student Diana (White British working class woman) aspired to continue doing STEM research but was stifled by her lack of self-confidence. She said, ‘I’m not especially talented… the theoretical side of physics is not really my skillset’, even though she recognised her own disciplinary competence. However, the idea of a research career seemed less attractive when she considered the limited opportunities in research:

I just hear so many stories about how difficult it is to get jobs in research now. I’m going to do a Masters somewhere else and specialise in something else that’s a lot more applied… that’s a lot more specific to getting a job, basically. And then go from there.

Similarly, biology student Katherine (British mixed heritage middle class woman) thought about an academic career in STEM but remained unconvinced as to whether it was the pathway for her. She shared her concerns that academics seem ‘very intense… they don’t really have a work life balance from what I can see’, citing an example of seeing her lecturer ‘leaving his lab at like 7 pm on a Sunday’. She saw academics as ‘people who are willing to devote a lot of time and effort and energy into their research… devote your life to it and work really long, hard hours’, a pathway which Katherine admits is becoming less attractive. Here, students may feel conflicted between their personal interest in STEM, especially in an academic career, and the reality and nature of the profession, which are less appealing in terms of work-life balance and financial remuneration, when compared to some non-STEM careers.

As the only medical student who expressed uncertainty about her future, Hafsa (British South Asian middle class woman) was initially hopeful and optimistic about being a doctor and ‘saving lives’. However, she was hesitant about a future in the medical field due to her negative experiences with some doctors. As Hafsa reflected:

I didn’t have the best experiences with doctors, and they all happened to be male… I feel like the way they speak to me might be more of the like, speaking down to someone rather than speaking to someone… I felt quite bullied in that sense [and] it’s clouding my judgement [about] the whole medical profession.

Hafsa’s experiences of gender inequality challenged her sense of identity and belonging in medicine. In particular, her experiences on work placements, which are central in medical training, raised doubts about her future as a doctor: ‘I was really struggling to see if I would fit in there if I would enjoy my time there’. As Hafsa has already completed 5 years of her medical degree, she decided to ‘give myself a couple of years in the NHS before I rule it out’, highlighting her uncertain future in STEM due to experiences of discrimination, despite her passion and interest in healthcare.

As can be seen, a degree in STEM, even medicine, does not guarantee a career in the discipline, even if students are interested and skilled. As science identity theory highlights, the importance of external factors, especially negative experiences and structural inequalities, can act as barriers to sufficient recognition by others. Such experiences can also damage their outcome expectations, a crucial part of future career planning as discussed in SCCT, which can challenge their career intentions in the short or medium-term.

## Discussion

This paper investigated the career intentions of under-represented students in STEM higher education. We identified three pathways, namely, driving into an STEM future, the end of the STEM road and a crossroads in STEM, to highlight the fragility of the STEM pipeline during the transition from undergraduate study to further study or work. In this section, we discuss the implications of our findings, revisiting the lenses of science identity and Social Cognitive Career Theory (SCCT).

For students who are committed to continuing their journey in STEM beyond undergraduate study, their aspirations are fuelled by interest in the discipline alongside a high degree of self-efficacy and relatively positive future outcome expectations (c.f. SCCT, see Lent et al., 2005). With the exception of engineering students, which merits further research, students from medicine and the applied sciences are particularly well represented on this pathway, which is unsurprising given these degrees are often vocational or have a directly related career. Training on the job, and work experience, are often part of these degree programme, which can strengthen the real-life applications of their learning and provide a preview of their future career in profession of their study (Jones et al., 2017). Some students, such as Michael, anticipated possible barriers as a result of their minoritised identities, but tried not to let anticipated barriers alter their career-related choices and maintained positive self-narrations about their future career paths. In short, the science identity of students who intended to drive into an STEM future appears strong, especially self-recognition as a science person (Carlone & Johnson, 2007), which was likely to be consolidated further if and when their STEM career plans were realised. However, such positive self-narration requires a considerable amount of identity work (Calabrese-Barton et al., 2013), which could lead to burnout if not given sufficient external recognition and support.

Perhaps the more concerning aspect of our findings was the prevalence of students who appeared to have reached the end of their STEM journey, with aspirations in non-STEM-related fields. These students are primed to exit the STEM pipeline, representing the ‘leak’ at the end of undergraduate education. It is often difficult for women and non-binary, ethnic minority and working class students to enrol in STEM degree programmes in the first place (Archer & DeWitt, 2017; Wong, 2016), but after acceptance these students continue to negotiate inequalities at university which can have a detrimental effect on their science identity and sense of belonging in STEM (Blackburn, 2017; Ong et al., 2018; Wong et al., 2021). Almost one in four students in our study plans to leave STEM after graduation and the main reason appears to be financial. Not only do students see non-STEM jobs, especially in finance-related careers as having greater and quicker economic returns, these opportunities also seem more plentiful, secure and accessible when compared to the STEM sector, especially in academia.

The apparent lack of engineering students in our study with an STEM career aspiration—a discipline with a direct route into professional employment—may be coincidental, but perhaps that also reflects how study options are structured in most UK universities, especially in STEM disciplines. In particular, students make university study decisions before entry—and disciplines, such as engineering do not often exist as a subject in school—but there is often limited flexibility for these STEM students to change their degrees after they start without a cost or time penalty. Furthermore, some STEM careers demand specific prior experiences or skillsets that might require further study or particular work experiences, which can be more difficult to find (than, say finance-related internships) or less rewarding economically (especially if such STEM internships are under-funded or unpaid). STEM careers in academia or research are perceived as less desirable because of their apparent heavy workload, limited career opportunities, and lack of work–life balance, especially in the initial phase.

SCCT suggests that appetite for STEM jobs is low, because the STEM field is seen as excessively competitive, with limited opportunities and economic rewards, weakening students’ outcome expectations and interest. For these undergraduates, STEM careers are ‘not for me’ and their identity in STEM would appear more pragmatic and extrinsic (Wong, 2016). Their engagement in STEM is now premised on the exchange value provided by the degree, more so than their initial interest or passion in the discipline, which appears to be supported by their taste of accomplishment elsewhere. For instance, many interviewees underwent successful work placements or internships in a non-STEM field, especially in business and finance, where their competences were validated and rewarded. These positive experiences reinforced their career intentions to depart from STEM, coupled with the symbolic value afforded by their universities and STEM degrees, whereby they appear to be in high demand from non-STEM graduate employers (Wakeham, 2016).

From a policy perspective, the enrolment of under-represented students in STEM degrees is a success, but their career intentions away from STEM merit further attention. On the one hand, the exchange value of STEM degrees is being realised, which would strengthen the discourse around STEM qualification as highly valuable in a wide range of careers. On the other hand, the departure of STEM graduates from STEM jobs, when governments and industries have concerns about shortage in STEM-skilled professionals (EMSI, 2018), might appear baffling and ironic. It is, of course, inevitable that some students leave the STEM field, but we need to be critical and sensitive about who stays and who goes, and whether there are patterns and structures of inequalities that appear to prevent or limit certain groups from further participation. The experience of Francesca, for example, illustrates how being very under-represented can shape career aspirations, and highlights the importance of greater inclusion and diversity, especially for under-represented students. The current dominance of men, especially White men, in the UK STEM workforce, particularly at senior levels (BSA, 2020; WISE, 2020), is still likely to play a role in the number of under-represented students considering as well as staying in an STEM career (Blackburn, 2017).

The third pathway we identified was the undecided, or those who were at a crossroads in their STEM future. Over one in three STEM undergraduates in our study were uncertain about their careers and these students included different disciplines and levels of study. Again, it is common for students to be open and flexible about their future, as higher education allows students time to develop their career intentions. However, if we wish to support students’ post-degree pathways, we need to facilitate and support their decision-making process. Given the rising importance of ‘graduate outcomes’—a metric that rates UK universities by undergraduate employability 18 months after degree completion—there is a growing emphasis on universities expanding the career support for their students (Hewitt, 2020). Yet, our students have raised concerns about the limited career advice from their tutors and departments, who seem more adept at providing opportunities for future academics in the specific discipline (see Molesworth et al., 2009). Understandably, the aims of these degrees are likely to be about mastery of the discipline, and thus the training of future experts in the degree subject, such as the example of Greg. However, students are likely to benefit from a broader range of career advice and opportunities, beyond the narrow route of STEM academia as advocated at the department level. While understandably tutors themselves may have limited experience in these broader STEM careers to support their students and this is beyond the remit (and workload) of their position, we suggest a closer collaboration between STEM departments and university careers services to help bring a wider portfolio of expertise and resources that are likely to be of use for undecided students (Ledwith, 2014). McGee and Bentley (2017) suggested that STEM career options ought to be broadened and embody the values of social justice, empathy and equity to appeal to a more diverse population, especially those from under-represented backgrounds.

From a strategic perspective, our undecided students would constitute a ready-made pool of potential STEM professionals who are trained with valuable STEM knowledge and skills. Their undecidedness can also be seen as an opportunity for different stakeholders in STEM to respond with targeted efforts and diverse opportunities for STEM undergraduates to reinvigorate their career aspirations in or from STEM. Much like the breadth of initiatives that aim to promote STEM as a viable study option beyond compulsory education (especially for under-represented students, such as girls, see Prieto-Rodriguez et al., 2020), a similar range of efforts may be needed for under-represented STEM undergraduates to strengthen their identities in STEM and facilitate a transition from undergraduate to STEM postgraduate courses or jobs. For instance, paid summer schools for particular under-represented students are common in STEM in the UK prior to university, but much less common at a postgraduate level. An expansion of such schemes would help provide paid and facilitated support for students’ development of science identities, skills, and experience. Our findings suggest that a strong sense of self and confidence in STEM would enable some students to counteract structural inequalities, such as (cis)sexism or racism, although we concur with Archer et al. (2021) in that the underlying problem is the field of STEM and how its structures and practices can systematically exclude or challenge certain social groups (e.g., Hafsa). Thus, it is important to highlight the role of STEM employers, as discussed by Smith and White (2019) who argued that employers’ recruitment strategies have an impact on pathways from STEM degrees into employment. We argue that STEM employers could provide more paid internship and graduate scheme opportunities to provide clear pathways and financially supported opportunities to gain relevant professional experience and ensure a diversity of candidates have access to these opportunities. This would also help divert STEM graduates from pursuing the lucrative and much clearer career pathways into finance and business. As such, while efforts to support students remain useful and practical, especially in the short-term, the more difficult but powerful approach would be to disrupt and equalise established practices and discourses across STEM education and employment, where more research is merited. Furthermore, future studies should also delve deeper into the data and experiences of the transition from STEM degree to STEM workforce among different under-represented groups.

## Conclusions

We are reminded that the STEM pipeline is far from secured or equitable. We conclude by reiterating the importance of fostering a diverse, inclusive and supportive learning environment that maximises the participation, strengths and potential of all students, especially those from under-represented backgrounds. Our data and the broader literature highlight that intersecting inequalities in STEM and society more generally can have a significant impact on students’ science identities and decisions around future career pathways. However, intersecting inequalities and under-representation are not deterministic; some students manage to persist down the STEM road and we can learn from the factors which facilitate their journeys. General efforts to address inequalities in STEM and society can disrupt barriers to STEM entry and ongoing participation. However, more tailored careers support and STEM identity development are also important, particularly external recognition of students’ STEM identities from academic staff, peers and potential STEM employers. The fostering of strong science identity throughout students’ STEM journeys can help to ensure robust disciplinary identities to withstand different barriers and setbacks.

Finally, the enthusiasm for non-STEM graduate schemes and other secure, well-paid graduate jobs should act as an encouragement for STEM employers to provide more supported routes into STEM employment, and for universities to provide clearer advice on how to navigate more complex and non-linear STEM career pathways to help manage students’ outcome expectations when unsure STEM futures discourage otherwise qualified and passionate STEM graduates. While we acknowledge that it is common for STEM students to pursue careers outside of STEM, and this is the right decision for some students, we must be wary that those who exit the STEM pipeline are not *forced off the road* by social inequalities and exclusions or unsupportive transition from graduation to graduate employment.

## Supplementary Information

**Additional file 1.** Interview Questions.

## Data Availability

Not applicable.
