# Supplementary material for: End of the road? The career intentions of under-represented STEM students in higher education
Source: Int J STEM Educ. 2022 Jul 30;9(1):51. doi: 10.1186/s40594-022-00366-8 (PMC9362640; doi:10.1186/s40594-022-00366-8)
Supplement: Supplementary file 1 — Additional file 1. Interview Questions. [file 40594_2022_366_MOESM1_ESM.docx]

Interview Questions:

- How are you today? Where are you right now?
- Tell me a little bit about yourself? Where do you live now? Did you live there prior to starting at university, or somewhere else? Who is in your family and what do they all do? Tell me a bit about your school – what kind of school was it, what did you study?
- *Check demographic information from the sign up survey*

*Subject/University:*

- Why did you decide to study X subject? Why did you decide to study at X university?
- Why did you decide to study now as opposed to in the past? [only for mature students]
- Do you work alongside your studies?
  - Type of job/hours worked
  - How do you balance studying/working?
  - Are you involved in any student societies or the students’ union? Which ones?
- Can you describe your first week at university? (How did you feel? Did you feel like you belonged/fitted in? Why? How does this compare to now?)
- Do you spend time with other students from university outside of class? Do you spend time with other students from your degree programme outside of class? Why?
- Did you stay in student accommodation/halls? What was this experience like?
- Which spaces and places are you most comfortable in on campus? Do you spend much time on campus? Why?
- How is your overall experience of university so far?
  - In terms of your academic experience
  - In terms of your social life
  - Can you tell me about a time when you felt a real sense of belonging at university?
  - Can you tell me about a time when you really didn’t feel like you belonged at university? For example, have you experience anything at University that was unpleasant or not quite what you expected?
  - Are you proud to be a student of your University? Why/why not? Do you feel like you fit in or belong here?

*Ideal Disciplinary Identity:*

- How are you doing so far in your degree? Do you enjoy it? How do you feel you are doing so far? Are you doing well?
- Can you describe a typical student from your degree programme/discipline?
- What makes an ideal student from your degree programme/discipline?
- How much do you think you fit these ideas of a typical or ideal student from your degree programme/discipline? Does this affect your experience of studying? If so, how does it affect you?

*Career aspirations and Ideal Professional Identity:*

- Are there particular careers or jobs associated with your degree programme?
- Is the career path into these careers or job clear? (If so, can you describe this path)
- Are there any opportunities in your programme of study that supports to work in this career/job?
- Describe a typical person in X career/job.
- Do you want to follow that career path or do that job in the future? Why? If no, what do you want to do/have you thought about careers?
- Do you think you could fit into that career path/job? Why? (Any barriers?).

*Closing Questions:*

- Do you think your experience as a student is different because you are a women/LGBTQ+/ ‘BAME’/first generation/working class/disabled/international/mature student? If yes, how is it different?
- Is this something you have talked about before? With friends, family, classmates, lecturers/tutors, or to anyone else? If so, where? If not, why not?
- Has talking about these things brought up anything surprising or brought up things you didn’t expect? Was there anything you thought I’d ask that I have not asked?
- Is there anything else you want to bring up that we haven’t covered yet?
- Identify three things that would improve the student experience at your university?
- Any other questions?
